# 

**Published:** 1898-04-02

**Authors:** 


					o
THE HOSPITAL. ? "The standard of highest purity."?Lancet. April 2nd, 1898.
CADBURY'S ^ wjr CADBURYS
cocoa '1 cocoa
ABSOLUTELY PURE. NO DRUGS OR ALKALIES USED.
ISi
"tt-^^fctiCtAacorv W"?TFBN Infirmary
No. 601. Vol. XXIY. C3S5JS
Saturday,
April 2nd, 1898.
[-antar^nTenu,] pMCB TW0PENCE

				

